# The Diagnostic and Prognostic Impact of Cardiovascular Magnetic Resonance in Ischemic Heart Disease: A Systematic Review

**DOI:** 10.3390/jcm15187196

**Published:** 2026-09-16

**Authors:** Andra-Maria Barota-Bebeșelea, Vasile Calin Arcas, Doru-Florian Cornel Moga, Anca Maria Fratila, Minodora Teodoru, Mihai Octavian Negrea, Ana-Maria Cristina Arcas, Ioan Manițiu

**Affiliations:** 1 Faculty of Medicine, Lucian Blaga University of Sibiu, 550169 Sibiu, Romania; andra.bebeselea@gmail.com (A.-M.B.-B.); anca.fratila@ulbsibiu.ro (A.M.F.); minodora.teodoru@ulbsibiu.ro (M.T.); ioanmanitiu@yahoo.com (I.M.); 2 Military Clinical Emergency Hospital of Sibiu, 550024 Sibiu, Romania; 3 Sibiu County Emergency Clinical Hospital, 550245 Sibiu, Romania; 4 Sibiu Municipal Clinical Hospital, 550245 Sibiu, Romania; 5 County Emergency Clinical Hospital Brașov, 500326 Brasov, Romania; arcascristina@yahoo.com; 6 Cardiology Practice “Dr. Manițiu Ioan”, 550245 Sibiu, Romania; 7 MedLife Polisano Hospital, 550253 Sibiu, Romania

**Keywords:** cardiovascular magnetic resonance, ischemic heart disease, stress perfusion, late gadolinium enhancement, microvascular obstruction, global longitudinal strain

## Abstract

**Background:** Ischemic heart disease (IHD) diagnosis is complicated by anatomical-functional dissociation, where morphological stenosis severity fails to reliably predict hemodynamic significance. This diagnostic gap necessitates a shift toward integrated physiological and structural phenotyping, a role increasingly fulfilled by Cardiovascular Magnetic Resonance (CMR). **Objective:** To systematically evaluate the incremental diagnostic and prognostic value of CMR across the clinical spectrum of IHD, defining its evidence-based integration into multimodal pathways, particularly for diagnostically indeterminate cases. **Methods:** A systematic review was conducted in accordance with the PRISMA guidelines. A structured literature search of PubMed, Web of Science, and Scopus retrieved peer-reviewed articles published between January 2021 and April 2026. Methodological quality was appraised using the Newcastle–Ottawa Scale and AMSTAR 2, while evidence certainty was evaluated via the GRADE framework. **Results:** Thirty studies were included in the qualitative synthesis. Current evidence demonstrates that stress perfusion CMR provides superior diagnostic accuracy compared with traditional tests (e.g., SPECT, stress ECG), although it may be outperformed by advanced CT-derived fractional flow reserve in specific post-CCTA cohorts. In indeterminate presentations such as MINOCA, CMR accurately reclassified the underlying etiology in approximately 68% of cases. Furthermore, multiparametric assessment—integrating late gadolinium enhancement (LGE), microvascular obstruction (MVO), and global longitudinal strain (GLS)—refined post-infarction short- and medium-term risk stratification beyond LVEF alone, though the independent prognostic value of MVO attenuates in long-term follow-up (>6 years). Emerging data support the non-inferiority of accelerated stress-only protocols and the utility of artificial intelligence-driven analytics. **Conclusions:** CMR is a critical modality for resolving anatomical-functional mismatches and providing robust risk stratification. However, evidence does not support its use as a universal first-line test due to operational costs, scan duration, and patient contraindications. Expanding its clinical impact relies strictly on standardizing protocols and adopting AI-assisted, accelerated sequences to mitigate logistical barriers.

## 1. Introduction

Ischemic heart disease (IHD) is the leading cause of global cardiovascular mortality, imposing significant socioeconomic burdens [1,2,3,4]. Its broad pathophysiological spectrum, from myocardial ischemia to sudden cardiac death, demands rapid, accurate diagnosis of coronary perfusion anomalies [5,6], as delays risk irreversible remodeling such as heart failure [7,8].

The heterogeneous and often atypical clinical spectrum of ischemic heart disease poses a central diagnostic challenge: well-documented anatomic–functional dissociation means that the morphological severity of epicardial stenosis does not reliably predict hemodynamic significance [9,10]. Anatomically severe lesions may lack meaningful hemodynamic impact, whereas moderate stenoses can unexpectedly cause significant impairment due to spatial complexity, eccentricity, and cumulative luminal resistance [11]. Consequently, exclusive anatomic evaluation remains inherently limited and insufficient for guiding revascularization or prognostic strategies. Accurate ischemic burden assessment requires simultaneous and comprehensive characterization of both structural anatomy and myocardial perfusion, enabling physiology-guided, individualized therapeutic decision-making [9,11].

The classical diagnostic algorithm for ischemic heart disease integrates functional and anatomical testing [12]. Resting electrocardiography (ECG) lacks sensitivity and stress ECG suffers from inadequate accuracy and inconclusive results due to comorbidities [13,14,15]. Consequently, stress ECG has become the preferred initial non-invasive approach [12,16].

Anatomically, coronary CT angiography excels in the detection of coronary artery disease (CAD), although its radiation exposure and iodinated contrast requirements restrict its use in certain patient profiles [13]. Furthermore, conventional anatomical CCTA may require subsequent functional validation because stenosis severity does not always correspond to lesion-specific ischemia [6]. This limitation primarily applies to conventional anatomical CCTA, whereas newer CT-derived fractional flow reserve techniques, including CT-FFR, enable non-invasive physiological assessment of lesion-specific ischemia and may partially overcome this anatomical–functional gap. Invasive coronary angiography remains the anatomical reference standard; however, its procedural risks and limited ability to evaluate the functional significance of coronary stenoses restrict its frontline role. This dissociation between anatomical severity and functional manifestations supports the contemporary shift toward multimodal imaging, including cardiovascular magnetic resonance [6,14].

European Society of Cardiology guidelines mandate integrated multimodal IHD imaging [2]. CCTA’s poor ischemic predictability in moderate stenoses [17] and frequent anatomical-functional mismatches [18] necessitate functional evaluation [19]. Functional modalities objectively quantify ischemia [20], detect microvascular dysfunction [21], and fulfill outcome trial mandates for physiological revascularization evidence [2]. Thus, pathways utilize Coronary CT Angiography (CCTA) for lower-risk and functional imaging for higher-risk scenarios [22]. Among functional modalities, CMR uniquely integrates anatomical and functional assessments to decisively guide interventions [23].

CMR is a non-invasive diagnostic modality that generates detailed cardiac images without ionizing radiation [24]. Its superior spatial and temporal resolution, combined with intrinsic soft-tissue contrast, has established CMR as a cornerstone in the evaluation of suspected or known ischemic heart disease [8,25,26].

In borderline or atypical ischemic heart disease presentations, it proves particularly valuable. Microvascular dysfunction-driven atypical angina is characterized through stress perfusion mapping and regional wall motion analysis [2,21,27], while silent ischemia is identified via LGE and cine CMR sequences [4]. It further differentiates true ischemic from non-ischemic etiologies, supporting risk stratification and therapeutic decisions in diagnostically ambiguous cases [28].

In diagnosing ischemic heart disease, CMR offers unique multiparametric advantages by combining anatomical and functional evaluation. Unlike coronary CT angiography’s morphological focus, CMR simultaneously assesses anatomy, ventricular function, and viability [29]. Compared with stress echocardiography, CMR provides superior, operator-independent reproducibility, as it remains unaffected by poor acoustic windows or high body mass index [21,26]. Furthermore, CMR serves as a safer, non-invasive alternative to invasive coronary angiography for risk stratification, avoiding periprocedural complications [3,30]. Crucially, unlike Single-Photon Emission Computed Tomography (SPECT) and Positron Emission Tomography (PET), CMR eliminates ionizing radiation while delivering superior spatial resolution, enabling the detection of minute subendocardial perfusion defects often missed by conventional scintigraphy [31].

These capabilities are delivered through dedicated CMR sequences. Cine MRI provides robust evaluation of ventricular function and wall motion, identifying functionally significant coronary artery disease [32]. Stress perfusion MRI assesses myocardial ischemia through gadolinium-based perfusion imaging [19]. Viability is assessed via LGE [4]. These techniques are augmented by T1 and T2 mapping, capable of detecting subtle myocardial damage, edema, and early fibrosis potentially missed by standard LGE [33].

Stress perfusion CMR evaluates myocardial ischemia using vasodilator stress and gadolinium contrast, directly visualizing subendocardial hypoperfusion through delayed signal intensity [34]. Advanced quantitative measurements of myocardial blood flow now allow objective differentiation between epicardial CAD and microvascular dysfunction (CMD) [22,27]. This modality demonstrates high diagnostic accuracy compared with invasive angiography [35]. Offering superior spatial resolution compared with SPECT, stress CMR serves as a first-line test to reliably detect ischemia and avoid unnecessary invasive procedures [36].

LGE CMR is the gold standard for myocardial viability, identifying necrotic tissue via gadolinium kinetics [37,38]. Transmural extent directs revascularization prognosis, where higher transmural scar burden predicts adverse outcomes [39,40,41,42]. Additionally, combining LGE with functional assessment refines long-term recovery predictions, particularly in intermediate transmurality segments [43,44].

Beyond its diagnostic origins, CMR delivers critical therapeutic guidance in ischemic heart disease [45]. By distinguishing reversible perfusion defects from irreversible fibrosis, it enables advanced non-invasive risk stratification and accurate prognostic anticipation [4,45]. This evaluation of myocardial viability directly alters clinical pathways, frequently refining medication strategies and guiding the necessity of percutaneous interventions or bypass surgeries [46]. CMR thus ensures evidence-based, cost-effective therapeutic pathways tailored to individual prognostic risk [45,46].

Despite its proven diagnostic value, CMR remains significantly underutilized in routine clinical practice, constrained by both technical and systemic barriers [47]. At the institutional level, high costs, prolonged acquisition times, and reliance on dedicated scanners and specialized expertise restrict availability predominantly to academic centers, limiting broader adoption [48,49,50,51,52]. At the patient level, lengthy and confined examinations frequently induce claustrophobia, occasionally necessitating sedation or scan cancellation [48,53,54], while legacy pacemakers, implantable cardioverter-defibrillators, and ferromagnetic implants impose direct safety contraindications [49,53,54]. Compounding these barriers, persistent discordance between American and European clinical guidelines complicates standardized integration into diagnostic pathways [55]. Consequently, urgent collaborative efforts are required to expand accessibility and establish CMR’s definitive role in routine ischemic heart disease management [52].

Nevertheless, the clinical adoption of CMR for ambiguous presentations remains limited by a lack of standardized algorithms. For patients with angina and non-obstructive coronary arteries, invasive testing has traditionally confirmed microvascular dysfunction (CMD) and its prognostic impact [56]. However, quantitative perfusion CMR and parametric mapping now enable the robust, non-invasive evaluation of microvascular dysregulation by objectively measuring myocardial blood flow [57]. Integrating stress CMR allows for reliable patient prescreening to identify specific endotypes, potentially averting the need for intracoronary instrumentation [58]. Furthermore, in complex syndromes like MINOCA, CMR-derived infarct characteristics and non-invasive microvascular assessments independently predict major adverse cardiovascular events [59]. Consequently, while emerging literature strongly supports CMR for indeterminate stenoses and CMD, rigorous integration into global clinical guidelines is urgently required to standardize therapeutic strategies [59].

Accordingly, this review pursued two interrelated objectives: first, to critically appraise the incremental diagnostic and prognostic value of CMR across the clinical spectrum of ischemic heart disease, from typical presentations to diagnostically indeterminate cases; and second, to define evidence-based criteria for its rational integration into contemporary multimodal imaging pathways, with specific focus on clinical scenarios where anatomical or functional modalities alone yield inconclusive results.

## 2. Materials and Methods

### 2.1. Study Design

The literature review component was conducted using a systematic and reproducible approach, adhering to the principles outlined by the PRISMA statement [60], in order to synthesize current evidence regarding the diagnostic accuracy, functional assessment, and prognostic value of CMR.

### 2.2. Search Strategy

The search strategy was independently developed and implemented by two reviewers (A.M.B.B. and A.M.C.A.), each assigned a predefined role to ensure methodological rigor and minimize selection bias. Specifically, each reviewer conducted the initial literature search in a different electronic database, while an additional reviewer was responsible for retrieving full-text articles. Duplicate records were subsequently identified and removed using standardized reference management procedures, facilitated by the web-based screening platform Rayyan, which enables efficient detection of duplicates and collaborative study selection while maintaining blinding between reviewers during the initial screening phases.

Following deduplication, all three reviewers independently screened titles and abstracts to determine preliminary eligibility. Full-text articles were then assessed through a multistage process: initially based on predefined inclusion and exclusion criteria, followed by a comprehensive evaluation of methodological quality and risk of bias using validated instruments such as the Newcastle–Ottawa Scale [61] for observational studies and AMSTAR 2 [62] for systematic reviews. The validity and consistency of extracted data were further appraised using structured data extraction frameworks and cross-verification methods, including inter-reviewer agreement analysis using Cohen’s kappa coefficient [63]. To ensure alignment across all stages of the review process, discrepancies between reviewers were resolved through consensus; in cases where agreement could not be reached, a third reviewer (I.M.) was consulted. The use of an odd number of reviewers was intentionally implemented to prevent decision ties, while formal agreement metrics and standardized protocols were employed to support the reliability and validity of the consensus process.

A grey literature search was not performed in this study. Therefore, only peer-reviewed articles indexed in the selected scientific databases were included. This represents a methodological limitation, as potentially relevant information may not have been identified.

### 2.3. Database Search

A comprehensive and structured database search was conducted across three major electronic platforms: PubMed, Web of Science, and Scopus, selected for their complementary coverage of biomedical and multidisciplinary literature. The search was restricted to studies published from January 2021 to April 2026, in order to capture the most recent and clinically relevant evidence reflecting advances in cardiac magnetic resonance imaging (CMR). A tailored search strategy was developed for each database, taking into account each platform’s specific indexing system and functionalities.

In PubMed, the strategy emphasized the use of controlled vocabulary through Medical Subject Headings (MeSH) [64], combined with free-text terms to maximize sensitivity. Boolean operators (AND, OR) were applied to refine the search [65].

For Web of Science, which does not rely on MeSH indexing, the search strategy was adapted to a keyword-based approach using topic fields (TS=), allowing simultaneous interrogation of titles, abstracts, and keywords. Here, emphasis was placed on broader term combinations and citation tracking features to identify highly cited and influential studies in the field.

In Scopus, a hybrid strategy was employed, combining keyword searches within TITLE-ABS-KEY fields with database-specific filters to enhance precision. Proximity operators and truncation (e.g., “ischemi*”, “magnetic resonance imag*”) were utilized to capture variations in terminology, thereby improving search sensitivity without compromising specificity.

Across all databases, Boolean logic was consistently applied to structure the search strategy, with combinations such as (“cardiac MRI” OR “CMR” OR “cardiovascular magnetic resonance”) AND (“ischemic heart disease” OR “coronary artery disease”) AND (“diagnostic accuracy” OR “prognosis” OR “viability”). This multi-layered and database-adapted approach ensured a comprehensive and reproducible identification of relevant literature, while minimizing the risk of selection bias and maximizing the retrieval of high-quality evidence.

The complete search strategy, including core keywords and database-specific Boolean combinations adapted for each platform, is presented in Table 1, Table 2, Table 3 and Table 4, ensuring transparency and reproducibility of the literature retrieval process.

### 2.4. Eligibility Criteria

Studies were selected based on predefined inclusion and exclusion criteria to ensure the relevance, quality, and applicability of the synthesized evidence.

Eligible studies met the following criteria:

1. Involved adult populations (≥18 years) with suspected or confirmed ischemic heart disease;
2. Evaluated the role of cardiac magnetic resonance imaging (CMR) in the diagnostic work-up, including ischemia detection, myocardial viability assessment, or prognostic stratification;
3. Reported clinically relevant outcomes such as diagnostic accuracy, functional assessment, or patient prognosis;
4. Were published in peer-reviewed journals in the English language within the predefined time frame (January 2021 to April 2026).

Studies were excluded if they:

1. Involved pediatric populations;
2. Did not specifically assess CMR or did not provide extractable data regarding its clinical utility;
3. Were non-original publications, including editorials, letters, conference abstracts without full text, or narrative reviews lacking methodological transparency;
4. Were not available in full text or published in languages other than English;
5. Contained insufficient methodological quality or high risk of bias, as determined during the quality assessment phase.

The review included original research studies with appropriate methodological designs, such as randomized controlled trials, prospective and retrospective cohort studies, and case–control studies. Diagnostic accuracy studies evaluating the performance of CMR against established reference standards (e.g., invasive coronary angiography or fractional flow reserve) were also considered eligible. Systematic reviews were included selectively for contextual discussion and cross-referencing but were not part of the primary data synthesis.

### 2.5. Study Selection Process

The study selection process followed a structured approach in accordance with the PRISMA recommendations [60,66]. In addition to the primary database search, a complementary manual search was performed by screening the reference lists and citations of the initially included articles, as well as identifying relevant studies mentioned within these sources. All newly identified records were subjected to the same screening and eligibility assessment protocols to ensure consistency and methodological rigor. The workflow was designed to ensure consistency and transparency across all stages of selection, maintaining independent assessment while allowing for structured resolution of discrepancies.

### 2.6. Data Extraction

Data extraction was performed using a standardized and predefined extraction template developed to ensure consistency, accuracy, and reproducibility across all included studies. The extraction process was conducted independently by the reviewers, with cross-verification to minimize errors and discrepancies.

The following variables were systematically collected: study characteristics (including author, year of publication, and study design); population details (sample size, demographic data, and clinical context); specific CMR techniques employed, such as stress perfusion imaging, LGE, and parametric mapping; and reported outcomes related to diagnostic performance and prognostic value, including measures of accuracy, ischemia detection, and risk stratification.

### 2.7. Quality and Risk of Bias Assessment

The methodological quality and risk of bias of the included studies were assessed using validated instruments selected according to study design, namely the Newcastle–Ottawa Scale for observational studies and AMSTAR 2 for systematic reviews [61,62]. The evaluation was performed independently by the reviewers, following standardized criteria to ensure consistency and reproducibility.

For observational studies, the Newcastle–Ottawa Scale assesses quality across three main domains: selection, comparability, and outcome/exposure. The selection domain includes criteria such as the representativeness of the exposed cohort (C1), the appropriateness of the non-exposed cohort (C2), and the method of exposure ascertainment (C3), as well as confirmation that the outcome of interest was not present at baseline (C4). The comparability domain (C5) evaluates whether studies adequately controlled for confounding factors through design or statistical adjustment, with up to two points awarded. The outcome/exposure domain includes assessment of how outcomes were measured (C6), whether the follow-up period was sufficient for outcomes to occur (C7), and the adequacy and completeness of follow-up (C8). This structured approach allows for a comprehensive evaluation of internal validity and potential bias in observational research [61,67].

Systematic reviews were assessed using AMSTAR 2, which evaluates methodological rigor across several critical domains. These include the clarity and appropriateness of the research question based on the PICO framework, the presence of a pre-registered protocol, the comprehensiveness of the literature search strategy, and the justification for excluding studies. Additional criteria assess whether the risk of bias in individual studies was adequately evaluated, whether appropriate statistical methods were used for data synthesis, and whether the potential impact of bias was considered when interpreting the results. Particular attention was also given to the inclusion of grey literature and the assessment of publication bias, as these factors may influence the overall validity of the evidence [62,68].

To complement these assessments, the overall strength and certainty of the evidence were evaluated using the GRADE framework. This approach considers multiple dimensions, including study limitations (risk of bias), consistency of findings across studies, directness of the evidence to the research question, precision of the estimates, and the likelihood of publication bias. Based on these criteria, the quality of evidence is graded from high to very low, providing a transparent and structured basis for interpreting the reliability of the synthesized findings [69].

### 2.8. Ethical Considerations and Reporting Standards

As this systematic review was based exclusively on previously published data and involved no direct participation of human subjects or collection of identifiable patient information, institutional ethics committee approval was not required. The ethical principles outlined in the Declaration of Helsinki were considered as a general framework [70]. The systematic review was conducted and reported in accordance with the PRISMA statement [60]. To ensure transparency and reproducibility, the review protocol was prospectively registered on the Open Science Framework [71] under the registration DOI 10.17605/OSF.IO/SKJV2.

## 3. Results

### 3.1. Databases Research Results

The systematic search of electronic databases yielded a total of 1148 records, identified through PubMed (*n* = 395), Scopus (*n* = 684), and Web of Science (*n* = 69). Prior to screening, 976 records were removed, including 848 duplicate records, 96 records identified as ineligible through automated screening tools, and 32 records excluded for other predefined reasons. Consequently, 172 records remained for title and abstract screening.

During the initial screening stage, 125 records were excluded because they did not meet the predefined eligibility criteria, leaving 47 reports that were sought for retrieval. Of these, three reports could not be retrieved, resulting in 44 full-text articles assessed for eligibility.

Following full-text assessment, four studies were excluded because they did not adequately address the predefined research question or the role of cardiac magnetic resonance in ischemic heart disease. An additional 14 studies were excluded because of insufficient methodological quality, defined as a Newcastle–Ottawa Scale score below the predefined threshold. No studies were excluded on the basis of AMSTAR 2 at this stage.

**Figure 1:**
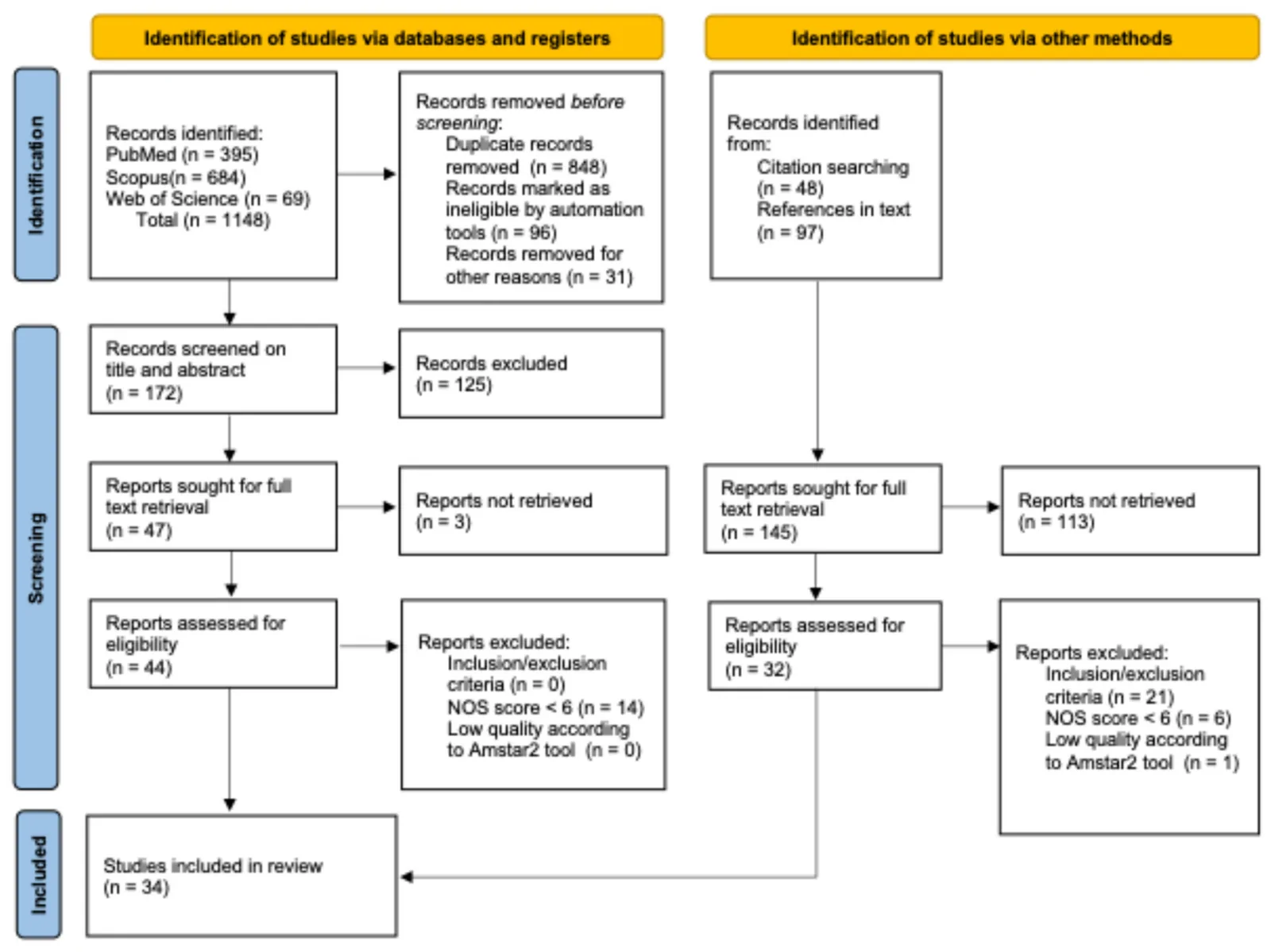
PRISMA flow chart illustrating the identification, screening, eligibility assessment, and inclusion of studies in the qualitative synthesis. The diagram was generated using the PRISMA 2020 online tool [72].

Consequently, 26 studies identified through database searching met the eligibility and methodological quality criteria and were included in the qualitative synthesis. The entire study selection workflow, including identification, screening, eligibility assessment, and final inclusion, is illustrated in Figure 1 (PRISMA flow diagram).

### 3.2. Other Sources’ Research Results

In addition to the database search, supplementary identification strategies were employed to ensure comprehensive coverage of the available literature. A total of 145 records were identified through alternative sources, including citation searching (*n* = 48) and references listed within previously selected articles (*n* = 97). These approaches were used to capture potentially relevant studies not retrieved through the primary database search.

All records identified through these additional methods were subjected to the same rigorous screening process. Of the 145 records, 113 reports were not retrieved, due not only to limited full-text availability or access restrictions, but also because a proportion of these records were found to be duplicates of studies already identified during the database search phase. As a result, 32 studies were successfully obtained and proceeded to full-text eligibility assessment.

During the eligibility phase, these studies underwent evaluation based on predefined inclusion and exclusion criteria, as well as methodological quality assessment. A total of 28 studies were excluded, including 21 studies that did not meet the inclusion criteria, such as lack of relevance to cardiac magnetic resonance imaging or absence of appropriate diagnostic or prognostic outcomes. Additionally, 6 studies were excluded due to insufficient methodological quality, defined as a score below 6 on the Newcastle–Ottawa Scale, and 1 study was excluded due to low quality according to the AMSTAR 2.

Following this selection process, four studies identified through other sources met the eligibility and methodological quality criteria and were included in the qualitative synthesis. Together with the 26 studies identified through database searching, a total of 30 studies were included in the final qualitative synthesis. The complete study selection process is presented in Figure 1.

### 3.3. Risk of Bias Assessment

The methodological quality of the included observational studies was evaluated using the Newcastle–Ottawa Scale, which assesses risk of bias across three domains: selection (C1–C4), comparability (C5), and outcome (C6–C8), with a maximum score of 9 points [61,67].

Within the selection domain, most studies demonstrated strong methodological quality, with all achieving full scores for representativeness (C1), exposure ascertainment (C3), and absence of outcome at baseline (C4), supporting both external validity and appropriate temporal relationships. However, variability was observed in C2, where several studies lacked clearly defined comparator groups, introducing potential selection bias.

In terms of comparability (C5), a large proportion of studies adequately adjusted for major confounders, although some showed only partial adjustment, leaving room for residual confounding. Within the outcome domain, outcome assessment (C6) and follow-up duration (C7) were generally appropriate across studies, while completeness of follow-up (C8) was less consistent, with some studies reporting insufficient follow-up, potentially introducing attrition bias.

Additionally, 21 studies were excluded during the methodological quality assessment: 20 observational studies due to Newcastle–Ottawa Scale scores below the predefined threshold and one systematic review due to low methodological quality according to AMSTAR 2.

Of these, 20 observational studies were excluded because their Newcastle–Ottawa Scale scores were below the predefined threshold (14 identified through database searching and 6 through other sources), while one systematic review identified through other sources was excluded due to low methodological quality according to AMSTAR 2.

Overall, the total NOS scores ranged from 6 to 9 points, as shown in Table 5, with the majority of studies classified as high quality (scores 7–9) and a smaller proportion as moderate quality (score = 6). No studies were categorized as low quality. The most common methodological limitations were related to the selection of appropriate comparator groups and the completeness of follow-up.

Taken together, these findings indicate that the included studies are characterized by a generally low to moderate risk of bias, with strong performance in key domains such as exposure ascertainment and outcome assessment. Consequently, the evidence base supporting the diagnostic and prognostic role of cardiac magnetic resonance imaging in ischemic heart disease can be considered methodologically robust, although the identified limitations should be taken into account when interpreting the results.

The methodological quality of the included systematic reviews and meta-analyses was assessed using the AMSTAR 2, with particular emphasis on the seven critical domains (Items 2, 4, 7, 9, 11, 13, and 15) [62,98,99]. The detailed evaluation of each study is summarized in Table 6.

Overall, most studies demonstrated moderate methodological quality, with consistent fulfillment of key domains such as risk of bias assessment (Item 9) and appropriate meta-analytical methods (Item 11) where applicable. However, variability was observed in several critical areas. Protocol registration (Item 2) was absent or only partially reported in some studies, representing a relevant methodological limitation. Similarly, justification for excluded studies (Item 7) and assessment of publication bias (Item 15) were inconsistently addressed, with most studies providing only partial information.

The adequacy of the literature search (Item 4) was generally satisfactory, while consideration of risk of bias in interpretation (Item 13) varied between studies, with only a subset fully integrating bias into their conclusions.

Based on these findings, the included reviews were classified as moderate to acceptable methodological quality, with the most common limitations related to incomplete reporting of exclusion criteria and insufficient evaluation of publication bias. Notably, one study was excluded due to critically low quality, failing to meet multiple critical AMSTAR 2 domains. Overall, while the included evidence is informative, these methodological limitations should be considered when interpreting the results.

The risk of bias analysis showed an overall favorable methodological profile, with most studies classified as low risk across the main domains, particularly selection and detection bias, supporting the reliability of the included evidence.

Some variability was observed in performance, attrition, and reporting bias, where several studies were rated as unclear, mainly due to incomplete reporting or limited adjustment for confounding factors.

**Figure 2:**
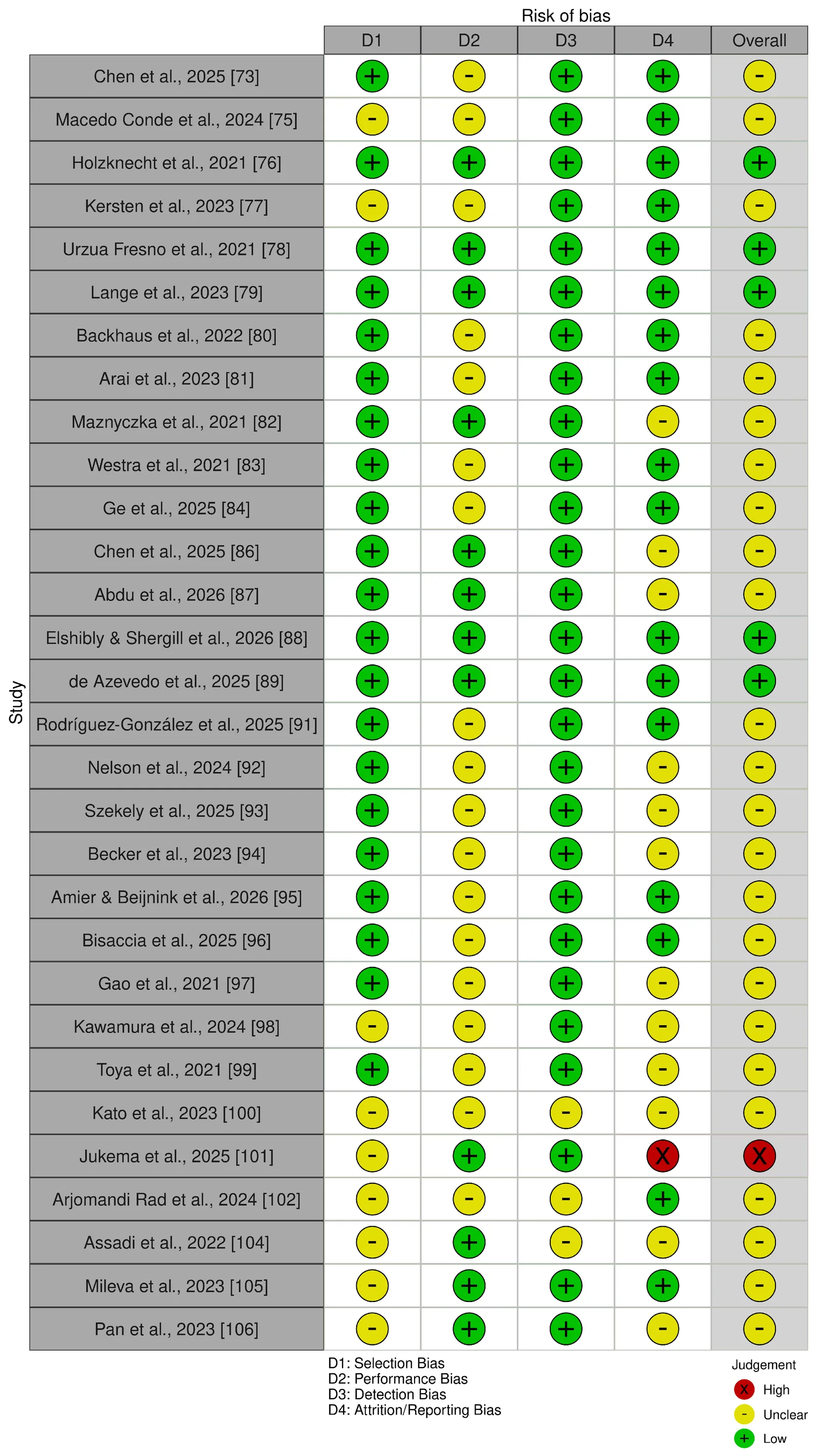
Traffic light plot illustrating the risk of bias assessment for each included study [73,75,76,77,78,79,80,81,82,83,84,86,87,88,89,91,92,93,94,95,96,97,98,99,100,101,102,104,105,106] across the evaluated domains. The figure was generated using the robvis online tool [107].

**Figure 3:**
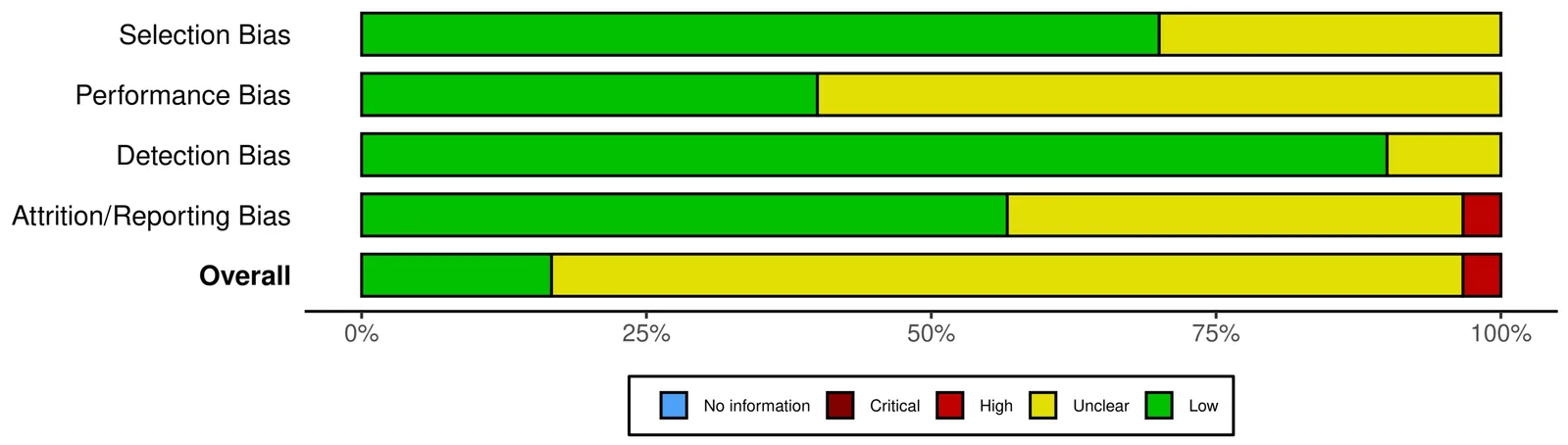
Summary plot showing the overall distribution of risk of bias judgments across the assessed domains. The figure was generated using the robvis online tool [107].

The detailed distribution of risk of bias across individual studies is presented in Figure 2 (traffic light plot), while the overall proportion of judgments across domains is summarized in Figure 3 (summary plot). Overall, although low risk of bias predominated, these findings indicate minor methodological limitations that should be considered when interpreting the results.

### 3.4. Synthesis of Findings

**Figure 4:**
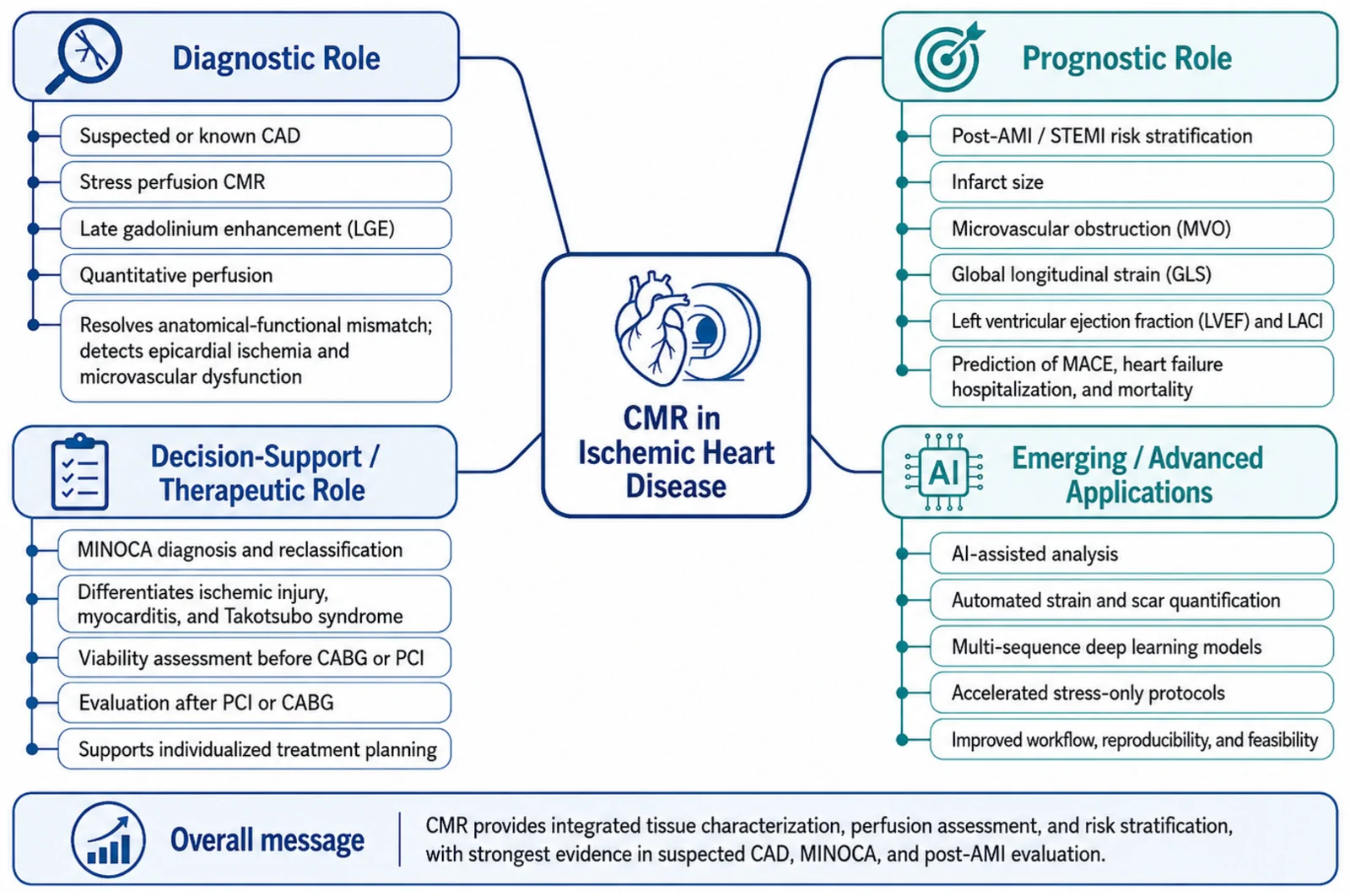
Evidence map summarizing the main diagnostic, prognostic, therapeutic, and emerging applications of CMR identified across the included studies.

The extracted studies collectively support the expanding clinical utility of cardiac magnetic resonance imaging in ischemic heart disease, not only as a diagnostic modality but also as a prognostic, tissue-characterizing, and decision-support tool. Across the included literature, CMR provided value beyond standard care by integrating functional assessment, myocardial perfusion, scar characterization, ventricular remodeling, and, in more recent studies, advanced parameters such as strain, quantitative perfusion, and artificial intelligence-assisted analysis; this can be seen in Figure 4.

#### 3.4.1. Diagnostic Value of CMR in Suspected or Known CAD

Several studies demonstrated that CMR improves the diagnostic evaluation of patients with suspected or established coronary artery disease, particularly when compared with conventional or anatomy-based approaches [80,82,86,90,93,101,102]. Stress perfusion CMR showed superior diagnostic performance to SPECT and exercise ECG in comparative studies [80,93]. In the CE-MARC subanalysis, Bisaccia et al. [93] reported that CMR had higher sensitivity, specificity, positive predictive value, negative predictive value, and AUC than exercise ECG, while also providing stronger prognostic discrimination for MACE. Similarly, Arai et al. [80] showed that stress perfusion CMR was superior to SPECT for AUC, specificity, accuracy, PPV, and NPV, supporting the role of CMR as a reliable first-line functional imaging test in patients with suspected CAD.

The superiority of CMR over purely anatomical assessment was further emphasized by Szekely et al. [90], who demonstrated that visual invasive coronary angiography had limited accuracy for detecting reduced myocardial perfusion when quantitative first-pass perfusion CMR was used as the functional reference. This finding highlights an important clinical concept: anatomical stenosis does not always correspond to functionally significant ischemia. Therefore, CMR adds value by identifying both epicardial and microvascular causes of ischemia, which may be missed by angiography alone.

In contrast, Westra et al. [82] reported that CT-QFR outperformed CMR in a selected post-CCTA population. However, this result should be interpreted cautiously, given the specific study pathway, CTA-based selection, and relatively low CMR sensitivity in that cohort. Rather than diminishing the role of CMR, this study suggests that the diagnostic yield of CMR may depend strongly on patient selection, protocol quality, and the clinical pathway in which it is used.

#### 3.4.2. CMR as a Tool for MINOCA Diagnosis and Reclassification

One of the strongest areas of evidence concerns MINOCA, where CMR consistently demonstrated a major diagnostic impact. In patients with myocardial infarction and non-obstructive coronary arteries, standard angiography often fails to identify the underlying cause. Macedo Conde et al. [74] showed that CMR established a diagnosis in 74.2% of NSTEMI patients with non-obstructive coronary arteries, differentiating true MINOCA from myocarditis and Takotsubo syndrome. Importantly, early CMR within 14 days improved diagnostic yield, supporting the need for timely imaging.

This finding is reinforced by the meta-analysis of Mileva et al. [105], which showed that CMR reclassified 68% of patients initially labelled as MINOCA and confirmed true ischemic MINOCA in only 22%. Similarly, Chen et al. [84] reported diagnostic reclassification in 81% of patients with suspected MINOCA. Taken together, these findings indicate that MINOCA should be considered a working diagnosis rather than a final diagnosis, and that CMR is essential for distinguishing ischemic from non-ischemic myocardial injury. However, this difference likely reflects heterogeneity between the studies included in the meta-analysis and the single-center cohort analyzed by Chen et al., rather than a true discrepancy in CMR diagnostic performance.

**Figure 5:**
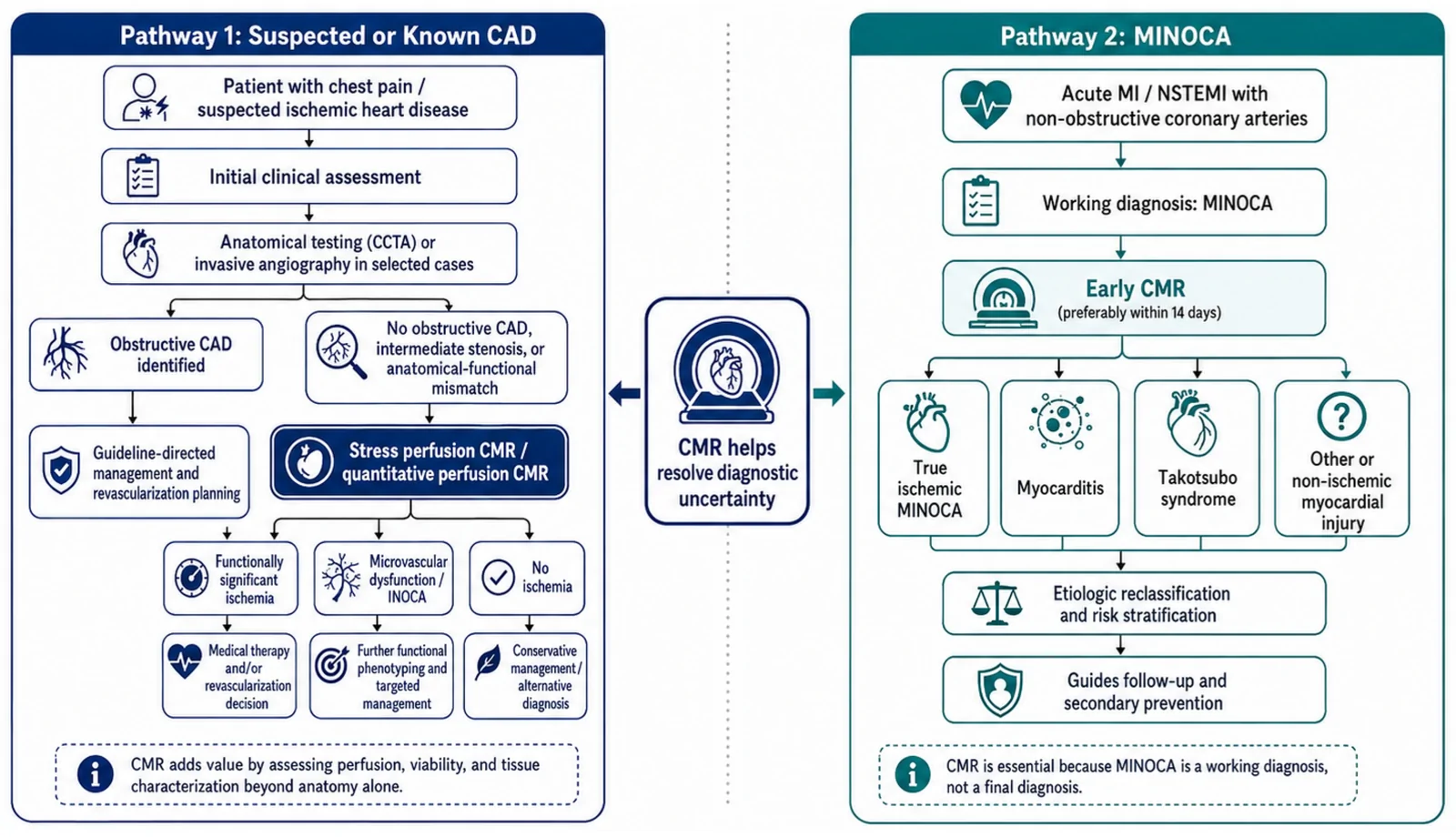
Proposed diagnostic impact of CMR in suspected CAD and MINOCA, illustrating how stress perfusion, LGE, and tissue characterization help resolve anatomical-functional mismatch and reclassify non-obstructive myocardial infarction.

Beyond diagnosis, CMR also adds prognostic information in MINOCA. Chen et al. [84] demonstrated that CMR-derived global longitudinal strain and left atrial reservoir strain independently predicted MACE in true MINOCA, while Abdu et al. [85] showed that infarct size by LGE-CMR, especially when combined with coronary microvascular dysfunction assessed by caIMR, identified patients with markedly higher MACE risk. These findings suggest that CMR is not only useful for etiological clarification but also for identifying high-risk MINOCA patients who may require closer follow-up and intensified secondary prevention, as shown in Figure 5.

#### 3.4.3. Prognostic Value After AMI and STEMI

The prognostic role of CMR after acute myocardial infarction is strongly supported by several studies. Traditional parameters such as infarct size, microvascular obstruction, and LVEF remain clinically important, but newer CMR-derived markers appear to refine risk stratification beyond conventional measures [73,75,78,79,81,83,92,94].

Chen et al. [84] demonstrated that a deep learning model integrating multi-sequence CMR data, including cine imaging, T2-STIR, and LGE, outperformed individual CMR parameters and standard clinical scores for predicting 2-year MACE after STEMI. This suggests that the combined interpretation of multiple CMR sequences captures complementary information on edema, infarction, MVO, and ventricular function.

Microvascular obstruction was repeatedly identified as an important post-STEMI marker. Maznyczka et al. [81] used CMR-defined MVO as the reference standard to validate thermodilution-derived temperature recovery time, showing that TRT was independently associated with MVO and predicted death, heart failure hospitalization, and MACE. However, Amier & Beijnink et al. [92] provided a more nuanced interpretation, showing that MVO was associated with mortality up to 6 years, but this effect was attenuated after adjustment for infarct size and LVEF and disappeared beyond 6 years. Therefore, MVO appears to be an important early and medium-term risk marker, but its independent long-term prognostic value may be limited.

**Figure 6:**
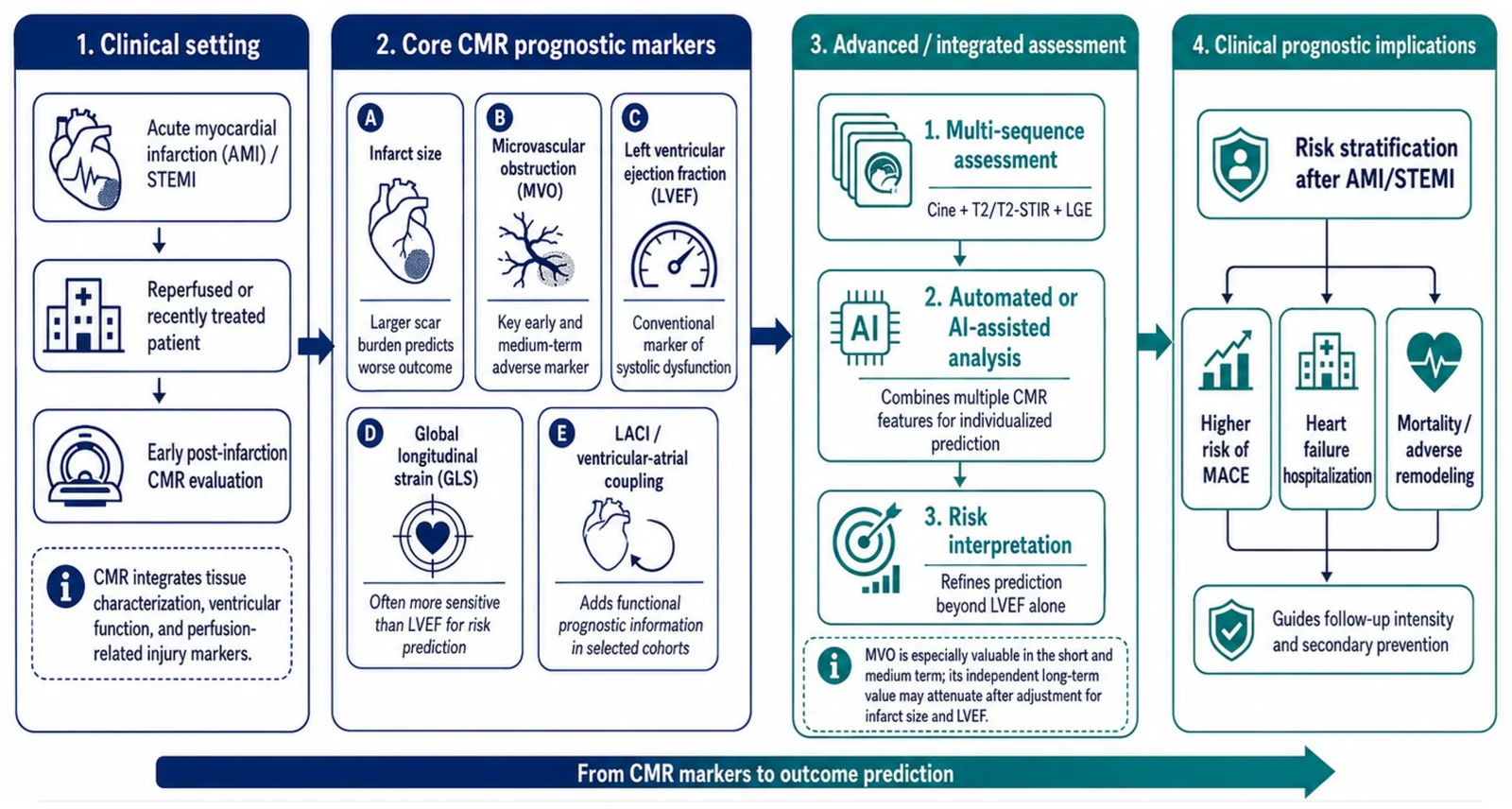
CMR-derived prognostic markers after acute myocardial infarction, showing how structural, microvascular, and functional parameters contribute to short-, medium-, and long-term risk stratification.

CMR-derived strain parameters also emerged as powerful prognostic tools. Holzknecht et al. [75] showed that GLS predicted MACE better than LVEF and remained independently associated with outcomes after adjustment for infarct size, MVO, and clinical variables. Backhaus et al. [79] extended this concept by demonstrating that automated GLS analysis showed similar prognostic accuracy to manual feature tracking, supporting its potential use in routine clinical workflows. Lange et al. [78] introduced the left atrioventricular coupling index (LACI), a simple CMR-derived marker of atrioventricular coupling and showed that it improved risk prediction beyond LVEF, especially in patients with LVEF ≤ 35%. However, when strain parameters were included, LACI lost independent significance, suggesting that strain may remain the more powerful functional prognostic marker. All this can be seen in Figure 6.

#### 3.4.4. CMR for Viability, Revascularization, and Post-Procedural Assessment

Several articles emphasized the value of CMR in guiding or evaluating revascularization. Arjomandi Rad et al. [103] showed that myocardial viability assessment was associated with improved survival after CABG, with LGE transmurality < 50% serving as an important marker of viable myocardium. This supports the use of CMR in identifying patients who may benefit from surgical revascularization, particularly when first-line tests are inconclusive.

Kersten et al. [76] evaluated CMR before and after CTO-PCI and showed that LGE-based viability assessment was useful for selecting patients eligible for revascularization. Post-procedural CMR demonstrated improved LVEF and reduced LV volumes, indicating positive remodeling. However, baseline CMR parameters did not predict symptomatic improvement, suggesting that viability imaging should be combined with clinical symptom assessment when selecting patients for CTO-PCI [76,79,103].

In the surgical setting, De Azevedo et al. [87] showed that LGE-CMR was useful for objectively detecting peri-procedural myocardial infarction after CABG. Residual SYNTAX score did not correlate with new LGE or biomarker release, but incomplete revascularization predicted higher 5-year MACE. This suggests that CMR provides accurate tissue-level assessment of myocardial injury, while long-term prognosis may also depend on procedural completeness and residual coronary disease burden [75,87].

#### 3.4.5. Quantitative Perfusion and Microvascular Disease

Quantitative perfusion CMR was particularly valuable for identifying ischemia beyond epicardial stenosis. Szekely et al. [90] demonstrated that many territories with reduced stress perfusion or myocardial perfusion reserve had no significant epicardial stenosis on invasive angiography, supporting the role of CMR in detecting microvascular dysfunction. Nelson et al. [89] also showed that women with INOCA had reduced myocardial perfusion reserve across LVEF groups, suggesting that microvascular dysfunction may be widespread and independent of conventional EF categories.

The prognostic relevance of quantitative perfusion was further supported by Pan et al. [106], who found that higher stress myocardial blood flow was associated with lower MACE risk across studies using CMR, PET/CT, and CT perfusion. Although this meta-analysis included mixed imaging modalities, it supports the concept that absolute myocardial blood flow provides prognostic information beyond anatomical coronary assessment.

Emerging protocols may further improve CMR feasibility. Elshibly & Shergill et al. [86] showed that an accelerated stress-only perfusion CMR protocol was non-inferior to standard stress-rest CMR for detecting significant CAD, while reducing scan time and contrast dose. This is clinically important because long acquisition times and limited access remain major barriers to widespread CMR adoption.

#### 3.4.6. Advanced Applications: AI, Automation, and Novel CMR-Derived Markers

The included studies show a clear trend toward more automated, quantitative, and AI-assisted CMR analysis. Backhaus et al. [79] demonstrated that automated GLS can replace manual feature tracking for post-AMI MACE prediction without loss of accuracy. Assadi et al. [104] also showed that AI-assisted CMR methods, including automated perfusion and volumetric/scar analysis, have prognostic value across cardiovascular diseases. Although the evidence base remains small and heterogeneous, these studies suggest that automated CMR analysis may improve reproducibility, reduce post-processing time, and support broader clinical implementation.

Chen et al. [84] further illustrate this direction by showing that deep learning integration of multi-sequence CMR data can outperform traditional risk scores and single CMR parameters after STEMI. These findings support the future role of CMR not only as an imaging test, but as a data-rich platform for individualized risk prediction.

#### 3.4.7. CMR as a Comparator or Reference Standard in Multimodality Imaging

Two studies used CMR as a reference or validation tool rather than as the primary intervention. Maznyczka et al. [81] used CMR-defined MVO to validate TRT as an invasive marker of microvascular reperfusion failure. Rodríguez-González et al. [88] used LGE-CMR to detect LV thrombus and quantify infarct size after STEMI, serving as an objective reference for cardioembolic risk assessment.

These studies reinforce the status of CMR as a reference standard for myocardial tissue characterization, infarct assessment, and certain structural complications after ischemic injury.

#### 3.4.8. Overall Interpretation

Overall, the extracted evidence indicates that CMR provides substantial added value in ischemic heart disease through three major mechanisms: first, by improving diagnosis through stress perfusion, LGE, quantitative perfusion, and multiparametric tissue characterization; second, by improving prognostic stratification through infarct size, MVO, strain, LACI, myocardial blood flow, and AI-derived markers; and third, by guiding clinical decision-making in complex scenarios such as MINOCA, post-STEMI risk stratification, viability assessment before revascularization, and evaluation after PCI or CABG [73,74,75,78,79,80,81,85,87,90,92,93,94,102,103,104,105,106].

The strongest evidence supports the use of CMR in suspected CAD, post-AMI risk stratification, and MINOCA diagnosis. Studies such as Bisaccia et al. [93], Arai et al. [80], Macedo Conde et al. [74], Mileva et al. [105], Chen et al. [73], Holzknecht et al. [75], Backhaus et al. [79], and Ge et al. [83] directly demonstrate clinically meaningful diagnostic or prognostic benefit. Emerging evidence from Elshibly & Shergill et al. [86], Assadi et al. [104], and Chen et al. [73] suggests that accelerated protocols, automation, and AI may overcome current limitations related to scan duration, expertise, and workflow integration [73,86,104].

However, limitations remain. Many studies were retrospective, single-center, or based on selected populations [73,74,76,77,83,85,89,90,94]. Protocol heterogeneity, variable scanner platforms, limited ethnic diversity, short follow-up in some studies, and inconsistent use of invasive reference standards such as FFR, OCT, or IMR limit direct comparability [73,74,78,79,80,83,85,86,93,101,102]. Despite these limitations, the overall evidence supports CMR as a comprehensive, radiation-free, and clinically impactful modality in the contemporary diagnostic and prognostic work-up of patients with suspected or known ischemic heart disease [75,78,79,81,83,106].

The main characteristics of the included studies, the CMR techniques used, the added value of CMR over standard care, reported outcomes, and acknowledged limitations are summarized in Table 7.

A condensed overview of the included studies, including study design, principal CMR method, and main result, is presented in Table 7. The complete extracted data, including the added value of CMR over standard care, detailed outcomes, and acknowledged limitations, are provided in Supplementary Table S1.

#### 3.4.9. Algorithmic Integration of CMR in IHD Care

Based on the synthesized evidence, CMR should be implemented as a targeted decision-support modality rather than as a universal first-line test. Its greatest clinical impact appears at three key stages of IHD care: first, after anatomical testing when coronary stenosis severity and functional ischemia are discordant or indeterminate; second, early after suspected MINOCA, where tissue characterization can distinguish true ischemic injury from myocarditis, Takotsubo syndrome, or other non-ischemic causes; and third, after AMI/STEMI or before revascularization, where infarct size, MVO, LVEF, GLS, and viability markers refine prognosis and guide follow-up or treatment planning.

### 3.5. Strength of Evidence Assessment

The strength of evidence was assessed using the GRADE framework, considering risk of bias, inconsistency, indirectness, imprecision, publication bias, effect magnitude, dose–response relationship, and residual confounding [69]. The detailed evaluation is presented in Table 8.

Overall, the certainty of evidence ranged from very low to high, with most studies providing low-to-moderate certainty evidence. Higher certainty ratings were assigned only when serious downgrading concerns were absent or when predefined GRADE upgrading criteria, such as a large effect magnitude or dose–response relationship, were explicitly met.

Lower certainty ratings were mainly assigned to studies with retrospective or single-center designs, small sample sizes, limited event numbers, heterogeneous populations, or incomplete standardization of CMR protocols. The most common reasons for downgrading were risk of bias and imprecision, whereas inconsistency and indirectness were less prominent in the core CMR studies.

Overall, the GRADE assessment supports a moderately strong evidence base for the clinical utility of CMR in ischemic heart disease, while also highlighting the need for further prospective, multicenter studies with standardized imaging protocols and longer follow-up.

GRADE judgments were applied consistently across studies. Observational evidence was initially rated as low certainty. A “Serious” concern resulted in downgrading by one level and a “Very Serious” concern by two levels. Upgrading was applied only when predefined GRADE criteria were met, including a large or very large effect, a dose–response gradient, or evidence that plausible residual confounding would reduce the observed effect. Multivariable adjustment, sample size, blinded assessment, and study design characteristics were not considered independent upgrading criteria.

### 3.6. Reliability and Validity of Data Extraction Assessment

Data extraction was performed using a predefined standardized template to ensure consistency, transparency, and reproducibility. Extracted variables were organized according to the FAIR principles (findable, accessible, interoperable, and reusable), allowing clear traceability and comparison across studies [108].

To assess reliability, reviewers independently extracted and cross-checked the data. Disagreements were resolved by consensus or, when necessary, by consultation with a third reviewer. Inter-reviewer agreement was quantified using Cohen’s kappa coefficient, particularly for categorical decisions such as study eligibility and quality assessment. Values above 0.80 were considered to indicate strong agreement [63]. As shown in Table 9, agreement between reviewer pairs ranged from strong to almost perfect, supporting the consistency and reproducibility of the review process.

## 4. Discussion

Cardiovascular Magnetic Resonance (CMR) integrates functional and anatomical data to diagnose ischemic heart disease (IHD) accurately. Clinical practice has shifted from strictly anatomical evaluations of the coronary bed toward assessing physiological impact. Historically, IHD diagnosis relied on the angiographic severity of epicardial stenoses. However, atherosclerotic morphology correlates poorly with true hemodynamic limitation. This discrepancy necessitates precise functional testing. CMR combines tissue characterization with functional metrics in a single examination. It detects basic ischemia and decodes underlying pathophysiology using regional ventricular function analysis, perfusion dynamics, and LGE for necrosis identification. Resolving anatomo-functional dissociation is now a prerequisite for therapeutic intervention. Contemporary revascularization and risk stratification models depend on the objective demonstration of ischemic burden and viability. CMR supplies the necessary diagnostic and prognostic evidence for these models.

Stress perfusion CMR achieves high sensitivity, specificity, and area under the curve (AUC) for detecting myocardial ischemia across epicardial and microvascular territories. This diagnostic precision stems from its exceptional spatial resolution. CMR detects isolated subendocardial ischemia. This early manifestation of the ischemic cascade is often missed by lower-resolution modalities. These physiological capabilities allow CMR to outperform traditional non-invasive tests like Single-Photon Emission Computed Tomography (SPECT) and exercise electrocardiography.

The diagnostic and prognostic performance of CMR aligns with the findings of landmark multi-center trials. Our systematic review corroborates the Clinical Evaluation of Magnetic Resonance Imaging in Coronary Heart Disease (CE-MARC) trial. CE-MARC established the superiority of CMR over SPECT for detecting obstructive coronary disease. CMR safely guides therapeutic decisions without excess adverse events. This mirrors the outcomes of trials like MR-INFORM. MR-INFORM demonstrated that a stress perfusion CMR strategy is non-inferior to fractional flow reserve (FFR)-guided invasive angiography and reduces unnecessary revascularizations [109]. Minor variations in diagnostic accuracy and prognostic thresholds exist between our aggregated data and these earlier trials. Shifts in population selection and baseline risk profiles explain these differences.

Methodological evolution also plays a role. Older trials used visual, qualitative assessments. We analyzed advanced, fully quantitative perfusion and multiparametric mapping techniques. Contemporary cohorts increasingly feature complex pathophysiologies like microvascular dysfunction. Modern quantitative CMR provides enhanced sensitivity for these conditions that traditional trials could not capture.

CMR challenges the traditional clinical paradigm equating epicardial stenosis with myocardial ischemia. Visual invasive coronary angiography (ICA) provides high-resolution anatomical data. However, it cannot quantify the physiological consequences of structural lesions. Modern CCTA paired with CT-FFR incorporates physiological data. Still, relying solely on pure anatomy without functional correlation overestimates ischemic burden and can lead to unnecessary interventions.

Anatomical-functional dissociation is problematic in borderline cases. In lesions of intermediate severity (50–70% stenosis), morphological appearance correlates poorly with true hemodynamic significance [110]. Patients frequently present with discordant results from prior non-invasive testing. A positive stress echocardiogram might contradict a negative CCTA. In these gray areas, clinical inertia or over-treatment are significant risks. CMR acts as the definitive tie-breaker. It maps the extent of ischemia and viability accurately. This dictates clinical management. CMR triages patients between conservative medical therapy and necessary revascularization to ensure optimal therapeutic allocation. CMR converts diagnoses of exclusion into definitive pathological findings for complex presentations.

Routine integration of CMR alters standard clinical pathways. Its high negative predictive value reduces purely diagnostic invasive coronary angiographies. This minimizes catheterizations in patients with non-obstructive coronary arteries. CMR also facilitates personalized patient management. It moves away from binary assessments of disease presence. Instead, it measures the exact ischemic burden by calculating the specific percentage of myocardium at risk. This perfusion quantification provides prognostic value. For example, higher stress myocardial blood flow correlates with a lower risk of MACE [106]. Precise quantification guides clinical decisions. Revascularization is reserved for patients with a demonstrated physiological need. Others are safely assigned to optimal medical therapy.

The prognostic value of specific markers shifts over time. Microvascular obstruction (MVO) loses its independent predictive power after six years when adjusted for infarct size and LVEF [92]. This temporal variation requires a multiparametric strategy. Clinicians use MVO for short-term risk evaluation and LGE scar size for long-term trajectories. Deep learning tools processing multi-sequence CMR data [73] and automated GLS systems [79] combine these variables. These computational models provide stable, long-term risk stratification exceeding the capacity of isolated clinical parameters.

Several limitations exist within the analyzed literature and this review. The synthesized studies exhibit heterogeneity regarding patient cohorts and baseline risk profiles. Several trials rely on small sample sizes. Methodological variability across centers introduces challenges in data comparability. This variability includes differences in magnetic field strengths, scanning sequences, and pharmacological stress protocols. Pragmatic, real-world barriers limit widespread CMR adoption. In routine clinical practice, limited scanner availability, high operational costs, and lengthy acquisition times constrain its utility. Patient-specific factors restrict application.

The modality requires sustained breath-holds and regular sinus rhythm for optimal image quality. It remains contraindicated in individuals with claustrophobia, advanced renal failure, or incompatible pacemakers. Logistical and clinical barriers prevent CMR from serving as a universal primary tool in all environments.

Cost-effectiveness represents an important criterion when discussing whether CMR could influence clinical pathways or future guideline recommendations. However, in the present review, this aspect should be addressed cautiously, because the included studies mainly focused on diagnostic accuracy, prognostic value, or patient reclassification, while formal health-economic data were not available in the searched literature. Therefore, a separate dedicated study would be required to assess the cost-effectiveness of CMR, including cost–benefit ratio, cost per correct diagnosis, and cost per cardiovascular event prevented.

To improve the practical interpretation of the findings, the main advantages and limitations of CMR in ischemic heart disease are summarized in Table 10.

Standardizing protocols and integrating novel technologies will address current barriers and expand clinical utility. Researchers are investigating whether multiparametric mapping (T1 and T2) can serve as a contrast-free alternative for diffuse myocardial tissue characterization. Concurrently, artificial intelligence (AI) and deep learning algorithms are altering CMR workflows. AI-driven automation reduces post-processing interpretation time and lowers the barrier for operator expertise. It enhances prognostic models by integrating complex, multi-sequence datasets like DeepSTEMI. Accelerated stress-only perfusion protocols cut scan times without sacrificing diagnostic accuracy [86]. This makes CMR accessible for routine, high-volume clinical use.

## 5. Conclusions

Cardiac Magnetic Resonance (CMR) provides an essential diagnostic mechanism for transitioning the evaluation of ischemic heart disease from pure anatomical imaging to integrated structural and physiological phenotyping. Current evidence confirms that CMR yields diagnostic accuracy superior to traditional functional tests in resolving anatomical-functional mismatches, and serves as a critical modality for reclassifying indeterminate syndromes such as MINOCA. Furthermore, it refines post-myocardial infarction risk stratification through the combined assessment of LGE, microvascular obstruction, and global longitudinal strain. However, the evidence does not support CMR as a universal first-line modality. Its clinical utility is currently optimized as a targeted intervention for diagnostically ambiguous cases and complex risk profiling, preventing unnecessary invasive procedures. Broad integration into routine clinical pathways remains hindered by operational costs, prolonged acquisition times, and absolute patient contraindications. Consequently, expanding the real-world impact of CMR depends largely on the validation and adoption of accelerated stress-only protocols and artificial intelligence-driven analytics to mitigate existing logistical constraints.

## Figures and Tables

**Table 1:** Core Keywords and Search Terms.

Concept	Keywords/Terms
Imaging modality	“cardiac MRI”, “CMR”, “cardiovascular magnetic resonance”
Disease	“ischemic heart disease”, “coronary artery disease”
Ischemia assessment	“myocardial ischemia”, “stress perfusion”
Tissue characterization	“late gadolinium enhancement”, “myocardial viability”
Clinical role	“diagnostic accuracy”, “prognosis”

**Table 2:** Search Strategy—PubMed.

Step	Search Query
1	“Magnetic Resonance Imaging”[MeSH] OR “cardiac MRI” OR “CMR”
2	“Myocardial Ischemia”[MeSH] OR “Coronary Artery Disease”[MeSH] OR “ischemic heart disease”
3	“diagnostic accuracy” OR “myocardial viability” OR “prognosis”
Final	#1 AND #2 AND #3
Filters	Humans, Adults, English, January 2021–April 2026

**Table 3:** Search Strategy—Web of Science.

Step	Search Query
1	TS = (“cardiac MRI” OR “CMR” OR “cardiovascular magnetic resonance”)
2	TS = (“ischemic heart disease” OR “coronary artery disease”)
3	TS = (“diagnostic accuracy” OR “myocardial viability” OR “prognosis”)
Final	#1 AND #2 AND #3
Filters	Article, English, 2021–2026

**Table 4:** Search Strategy—Scopus.

Step	Search Query
1	TITLE-ABS-KEY (“cardiac MRI” OR “CMR” OR “cardiovascular magnetic resonance”)
2	TITLE-ABS-KEY (“ischemic heart disease” OR “coronary artery disease”)
3	TITLE-ABS-KEY (“diagnostic accuracy” OR “myocardial viability” OR “prognosis”)
Final	#1 AND #2 AND #3
Filters	Article, English, 2021–2026

**Table 5:** Newcastle–Ottawa Scale (NOS) Assessment of Methodological Quality of Included Studies.

Author, Year	C1	C2	C3	C4	C5	C6	C7	C8	Total Score
Chen et al., 2025 [73]	1	1	1	1	1	1	1	1	8
Macedo Conde et al., 2024 [74]	1	0	1	1	1	1	1	1	7
Holzknecht et al., 2021 [75]	1	1	1	1	2	1	1	1	9
Kersten et al., 2023 [76]	1	0	1	1	1	1	1	1	7
Urzua Fresno et al., 2021 [77]	1	1	1	1	2	1	1	1	9
Lange et al., 2023 [78]	1	1	1	1	2	1	1	1	9
Backhaus et al., 2022 [79]	1	1	1	1	1	1	1	1	8
Arai et al., 2023 [80]	1	1	1	1	1	1	1	1	8
Maznyczka et al., 2021 [81]	1	1	1	1	2	1	1	0	8
Westra et al., 2021 [82]	1	1	1	1	1	1	1	1	8
Ge et al., 2025 [83]	1	1	1	1	1	1	1	1	8
Chen et al., 2025 [84]	1	1	1	1	2	1	1	0	8
Abdu et al., 2026 [85]	1	1	1	1	2	1	1	0	8
Elshibly & Shergill et al., 2026 [86]	1	1	1	1	2	1	1	1	9
de Azevedo et al., 2025 [87]	1	1	1	1	2	1	1	1	9
Rodríguez-González et al., 2025 [88]	1	1	1	1	1	1	1	1	8
Nelson et al., 2024 [89]	1	1	1	1	1	1	0	0	6
Szekely et al., 2025 [90]	1	1	1	1	1	1	0	0	6
Becker et al., 2023 [91]	1	1	1	1	1	1	1	0	7
Amier & Beijnink et al., 2026 [92]	1	1	1	1	1	1	1	1	8
Bisaccia et al., 2025 [93]	1	1	1	1	1	1	1	1	8
Gao et al., 2021 [94]	1	1	1	1	1	1	1	0	7
Kawamura et al., 2024 [95]	0	1	1	1	1	1	1	0	6
Toya et al., 2021 [96]	1	1	1	1	1	1	1	0	7

C1—Degree to which the exposed cohort reflects the target population (0–1 point); C2—Appropriateness of the selection of the non-exposed cohort (0–1 point); C3—Method used to determine exposure (0–1 point); C4—Confirmation that the outcome of interest was not present at baseline (0–1 point); C5—Comparability of study groups based on design or statistical adjustment for confounders (0–2 points); C6—Method of outcome assessment (0–1 point); C7—Sufficiency of the follow-up period for outcomes to be observed (0–1 point); C8—Completeness and adequacy of follow-up of the study population (0–1 point) [61,67,97].

**Table 6:** Methodological Quality Assessment of Systematic Reviews Using AMSTAR 2 [100].

	Author, Year	Kato et al., 2023 [101]	Jukema et al., 2025 [102]	Arjomandi Rad et al., 2024 [103]	Assadi et al., 2022 [104]	Mileva et al., 2023 [105]	Pan et al., 2023 [106]
Critical Domains							
Protocol registered before commencement (Item 2)		Partial Yes	Yes	Partial Yes	Yes	Yes	Yes
Adequacy of the literature search (Item 4)		Yes	Yes	Yes	Partial Yes	Yes	Yes
Justification for excluding studies (Item 7)		No	Partial Yes	Partial Yes	Partial Yes	Partial Yes	Partial Yes
Risk of bias assessment of included studies (Item 9)		Yes	Yes	Yes	Yes	Yes	Yes
Appropriateness of meta-analysis methods (Item 11)		Yes	Yes	Yes	Yes	Yes	Yes
Consideration of risk of bias in interpretation (Item 13)		Partial Yes	Yes	Partial Yes	Partial Yes	Yes	Yes
Assessment of publication bias (Item 15)		Partial Yes	No	Yes	Partial Yes	Yes	Partial Yes

**Table 7:** Condensed Summary of the Design, CMR Methods, and Main Results of the Included Studies.

Author, Year	Design and CMR Method	Key Finding
Chen et al., 2025 [73]	Prospective cohort; multiparametric CMR + AI	DeepSTEMI improved 2-year MACE prediction after STEMI.
Macedo Conde et al., 2024 [74]	Retrospective cohort; cine, T2-STIR, LGE	CMR diagnosed 74.2% of MINOCA presentations; early imaging improved yield.
Kato et al., 2023 [101]	Systematic review; whole-heart coronary MRA	3.0-T contrast MRA had the highest CAD diagnostic accuracy.
Jukema et al., 2025 [102]	Meta-analysis; stress-perfusion CMR	CMR outperformed SPECT and showed better specificity than CCTA.
Holzknecht et al., 2021 [75]	Prospective cohort; feature-tracking CMR	GLS predicted MACE better than LVEF after STEMI.
Kersten et al., 2023 [76]	Prospective cohort; LGE before/after CTO-PCI	CTO-PCI improved ventricular function, but baseline CMR did not predict symptoms.
Urzua Fresno et al., 2021 [77]	Retrospective cohort; LV volumetry and LGE	LVEF and LV mass predicted ICD shock or death.
Lange et al., 2023 [78]	Multicentre cohort; LACI and strain	LACI predicted 12-month MACE, but strain was stronger.
Backhaus et al., 2022 [79]	Multicentre cohort; automated feature tracking	Automated GLS matched manual analysis and predicted MACE.
Arai et al., 2023 [80]	Multicentre trial; stress-perfusion CMR	CMR was diagnostically superior to SPECT for CAD.
Arjomandi Rad et al., 2024 [103]	Meta-analysis; CMR viability assessment	Viable myocardium predicted better survival after CABG.
Maznyczka et al., 2021 [81]	Derivation/validation cohort; LGE-defined MVO	TRT identified MVO and predicted adverse outcomes.
Westra et al., 2021 [82]	Prospective substudy; CMR after positive CCTA	CT-QFR outperformed CMR in this selected pathway.
Ge et al., 2025 [83]	Multicentre cohort; stress CMR and LGE	Ischaemia and scar independently predicted hard cardiovascular events.
Chen et al., 2025 [84]	Retrospective cohort; multiparametric CMR in MINOCA	CMR reclassified 81%; GLS and LA strain predicted MACE.
Abdu et al., 2026 [85]	Retrospective cohort; CMR + caIMR	Large infarction plus CMD identified highest-risk MINOCA patients.
Elshibly & Shergill et al., 2026 [86]	Randomised diagnostic study; stress-only CMR	Accelerated CMR was non-inferior and shortened scans by ~24 min.
De Azevedo et al., 2025 [87]	Prospective cohort; LGE before/after CABG	Incomplete revascularisation predicted higher 5-year MACE.
Assadi et al., 2022 [104]	Systematic review; AI-assisted CMR	AI-CMR showed prognostic value across multiple cardiovascular conditions.
Mileva et al., 2023 [105]	Meta-analysis; multiparametric CMR in MINOCA	CMR reclassified 68% and confirmed true MINOCA in 22%.
Rodríguez-González et al., 2025 [88]	Prospective cohort; LGE and LV thrombus	CMR detected LV thrombus in 19% after STEMI.
Nelson et al., 2024 [89]	Cross-sectional study; perfusion CMR in INOCA	Reduced perfusion reserve occurred across LVEF groups.
Szekely et al., 2025 [90]	Diagnostic study; quantitative perfusion CMR	Perfusion mapping distinguished epicardial from microvascular ischaemia.
Becker et al., 2023 [91]	Retrospective cohort; cine and LGE	Midwall LGE predicted ventricular arrhythmias.
Amier & Beijnink et al., 2026 [92]	Pooled cohort; long-term MVO assessment	MVO predicted mortality up to 6 years, but not thereafter.
Pan et al., 2023 [106]	Meta-analysis; quantitative myocardial blood flow	Higher stress blood flow was associated with lower MACE risk.
Bisaccia et al., 2025 [93]	Post hoc trial analysis; stress CMR	CMR outperformed exercise ECG and strongly predicted MACE.
Gao et al., 2021 [94]	Retrospective cohort; acute STEMI CMR	Higher SYNTAX score predicted larger infarction and less salvage.
Kawamura et al., 2024 [95]	Phase I trial; LGE after ADSC + CABG	ADSC therapy reduced scar and improved LV function.
Toya et al., 2021 [96]	Retrospective cohort; CMR-confirmed non-obstructive ischaemia	Resistive reserve ratio predicted long-term mortality.

**Table 8:** GRADE Assessment of the Certainty of Evidence Across Included Studies.

Study (Author, Year)	Initial Rating	Limitations (RoB)	Inconsistency	Indirectness	Imprecision	Publication Bias	Effect Magnitude	Dose- Response	Residual Confounders	Final Grade
Chen et al., 2025 [73]	Observational = Low	Serious	Not Serious	Not Serious	Not Serious	Unlikely	Large	N/A	AI model adjusted for all CMR	LOW
Macedo Conde et al., 2024 [74]	Observational = Low	Serious	Not Serious	Not Serious	Serious	Unlikely	Large	N/A	N/A	MODERATE
Kato et al., 2023 [101]	SR/MA = High	Serious	Serious	Not Serious	Not Serious	Likely	Small/None	N/A	N/A	MODERATE
Jukema et al., 2025 [102]	SR/MA = High	Serious	Not Serious	Serious	Not Serious	Likely	Large	N/A	N/A	MODERATE
Holzknecht et al., 2021 [75]	Observational = Low	Not Serious	Not Serious	Not Serious	Serious	Unlikely	Large	N/A	Not demonstrated	LOW
Kersten et al., 2023 [76]	Observational = Low	Serious	Not Serious	Not Serious	Serious	Unlikely	Small/None	N/A	N/A	LOW
Urzua Fresno et al., 2021 [77]	Observational = Low	Not Serious	Not Serious	Not Serious	Serious	Unlikely	Small/None	N/A	Not demonstrated	VERY LOW
Lange et al., 2023 [78]	Observational = Low	Not Serious	Not Serious	Not Serious	Serious	Unlikely	Large	N/A	Not demonstrated	LOW
Backhaus et al., 2022 [79]	Observational = Low	Not Serious	Not Serious	Not Serious	Not Serious	Unlikely	Large	N/A	Not demonstrated	MODERATE
Arai et al., 2023 [80]	Observational = Low	Serious	Not Serious	Serious	Not Serious	Unlikely	Large	N/A	independent blinded core labs	LOW
Arjomandi Rad et al., 2024 [103]	SR/MA = High	Serious	Not Serious	Not Serious	Serious	Unlikely	Large	N/A	N/A	MODERATE
Maznyczka et al., 2021 [81]	Observational = Low	Not Serious	Not Serious	Not Serious	Serious	Unlikely	Large	MVO extent increases	multivariable adjustment	HIGH
Westra et al., 2021 [82]	Observational = Low	Serious	Not Serious	Serious	Serious	Unlikely	Small/None	N/A	Not demonstrated	VERYLOW
Ge et al., 2025 [83]	Observational = Low	Not Serious	Not Serious	Not Serious	Not Serious	Unlikely	Large	ischaemia severity dose–response	Fine-Gray competing risk multivariable model	HIGH
Chen et al., 2025 [84]	Observational = Low	Serious	Not Serious	Not Serious	Serious	Unlikely	Small/None	N/A	N/A	MODERATE
Abdu et al., 2026 [85]	Observational = Low	Serious	Not Serious	Not Serious	Serious	Unlikely	Large	N/A	N/A	MODERATE
Elshibly & Shergill et al., 2026 [86]	Diagnostic study (RCT design)	Not Serious	Not Serious	Not Serious	Not Serious	Unlikely	N/A	N/A	N/A	HIGH
De Azevedo et al., 2025 [87]	Observational = Low	Serious	Not Serious	Not Serious	Serious	Unlikely	Large	N/A	N/A	MODERATE
Assadi et al., 2022 [104]	SR of observational studies = Low start	Very Serious	Very Serious	Serious	Very Serious	Likely	N/A	N/A	N/A	MODERATE
Mileva et al., 2023 [105]	Observational SR = Low	Serious	Not Serious	Not Serious	Serious	Likely	Large	N/A	N/A	MODERATE
Rodríguez-González et al., 2025 [88]	Observational = Low	Serious	Not Serious	Not Serious	Serious	Unlikely	Large	continuous RT-risk gradient	N/A	MODERATE
Nelson et al., 2024 [89]	Observational = Low	Serious	Not Serious	Serious	Serious	Unlikely	Small/None	N/A	N/A	MODERATE
Szekely et al., 2025 [90]	Observational = Low	Serious	Not Serious	Not Serious	Serious	Unlikely	N/A	N/A	N/A	MODERATE
Becker et al., 2023 [91]	Observational = Low	Serious	Not Serious	Not Serious	Not Serious	Unlikely	Large	N/A	N/A	MODERATE
Amier & Beijnink et al., 2026 [92]	Observational = Low	Serious	Not Serious	Not Serious	Not Serious	Unlikely	N/A	N/A	N/A	MODERATE
Pan et al., 2023 [106]	SR/Meta- analysis = High	Serious	Serious	Serious	Serious	Likely	N/A	N/A	N/A	MODERATE
Bisaccia et al., 2025 [93]	Observational (post hoc) = Low	Serious	Not Serious	Not Serious	Not Serious	Unlikely	Large	N/A	N/A	MODERATE
Gao et al., 2021 [94]	Observational = Low	Serious	Not Serious	Not Serious	Serious	Unlikely	Small/None	N/A	N/A	LOW
Kawamura et al., 2024 [95]	RCT = High	Very Serious	Serious	Serious	Very Serious	Unlikely	N/A	N/A	N/A	LOW
Toya et al., 2021 [96]	Observational = Low	Serious	Not Serious	Not Serious	Not Serious	Unlikely	Small/None	N/A	N/A	VERY LOW

**Table 9:** Inter-Reviewer Agreement During Study Selection and Data Extraction Using Cohen’s Kappa Statistic.

Reviewer Pair	Number of Assessed Items	Agreed Items	Disagreed Items	Cohen’s Kappa Value	Interpretation
A.M.B.B. & A.M.C.A.	38	34	4	0.82	Strong agreement
A.M.B.B. & I.M.	38	35	3	0.87	Strong agreement
A.M.C.A. & I.M.	39	37	2	0.91	Almost perfect agreement

**Table 10:** Main advantages and limitations of CMR in ischemic heart disease.

Advantages	Limitations
Multiparametric assessment	Higher cost
No ionizing radiation	Limited availability
Detects ischemia	Longer scan time
Assesses scar, edema, viability	Requires expert interpretation
Helps reclassify MINOCA	Contraindications in some patients
Improves post-AMI/STEMI risk stratification	Gadolinium limits in renal disease
Supports revascularization planning	Advanced protocols not fully standardized
AI/accelerated protocols may improve workflow	Not a universal first-line test

## Data Availability

The research was registered at the Open Science Framework (OSF) and can be found under registration DOI 10.17605/OSF.IO/SKJV2. All the metadata was uploaded and is available under the registration code osf.io/a9gdy.

## Supplementary Materials

The following supporting information can be downloaded at: https://www.mdpi.com/article/10.3390/jcm15187196/s1, Table S1: PRISMA 2020 Checklist.

## Abbreviations

The following abbreviations are used in this manuscript:

ADSC	Adipose-derived stem cells
AI	Artificial Intelligence
AMI	Acute Myocardial Infarction
AMSTAR 2	A Measurement Tool to Assess Systematic Reviews 2
AUC	Area Under the Curve
bSSFP	Balanced Steady-State Free Precession
CABG	Coronary Artery Bypass Grafting
caIMR	Catheter-based Angiography-derived Index of Microcirculatory Resistance
CAD	Coronary Artery Disease
CCTA	Coronary Computed Tomography Angiography
CE-MARC	Clinical Evaluation of Magnetic Resonance Imaging in Coronary Heart Disease (trial)
CFR	Coronary Flow Reserve
CMD	Coronary Microvascular Dysfunction
CMR	Cardiovascular Magnetic Resonance
CT	Computed Tomography
CT-FFR/CT-QFR	CT-derived Fractional Flow Reserve/CT-derived Quantitative Flow Ratio
CTO-PCI	Chronic Total Occlusion–Percutaneous Coronary Intervention
DSE	Dobutamine Stress Echocardiography
ECG	Electrocardiography/Electrocardiogram
ECV	Extracellular Volume
EF	Ejection Fraction
ESC	European Society of Cardiology
EuroCMR	European Cardiovascular Magnetic Resonance Registry
FAIR	Findable, Accessible, Interoperable, Reusable
FFR	Fractional Flow Reserve
GCS	Global Circumferential Strain
GLS	Global Longitudinal Strain
GRADE	Grading of Recommendations Assessment, Development and Evaluation
GRS	Global Radial Strain
ICA	Invasive Coronary Angiography
ICD	Implantable Cardioverter-Defibrillator
IHD	Ischemic Heart Disease
IMR	Index of Microcirculatory Resistance
INOCA	Ischemia with Non-Obstructive Coronary Arteries
IVUS	Intravascular Ultrasound
LA	Left Atrial/Left Atrium
LACI	Left Atrioventricular Coupling Index
LAS	Left Atrial Strain
LGE	Late Gadolinium Enhancement
LV	Left Ventricle/Left Ventricular
LVEF	Left Ventricular Ejection Fraction
LVEDVI	Left Ventricular End-Diastolic Volume Index
LVESVI	Left Ventricular End-Systolic Volume Index
MACE	Major Adverse Cardiovascular Events
MAPSE	Mitral Annular Plane Systolic Excursion
MBF	Myocardial Blood Flow
MeSH	Medical Subject Headings
MINOCA	Myocardial Infarction with Non-Obstructive Coronary Arteries
MR-INFORM	Magnetic Resonance Perfusion Imaging-Guided Management of Patients with Stable Coronary Artery Disease (trial)
MPS	Myocardial Perfusion Scintigraphy
MPRI	Myocardial Perfusion Reserve Index
MRA	Magnetic Resonance Angiography
MRI	Magnetic Resonance Imaging
MVO	Microvascular Obstruction
NOCAD	Non-Obstructive Coronary Artery Disease
NOS	Newcastle–Ottawa Scale
NPV	Negative Predictive Value
NSTEMI	Non-ST-Elevation Myocardial Infarction
OCT	Optical Coherence Tomography
OSF	Open Science Framework
PCI	Percutaneous Coronary Intervention
PET	Positron Emission Tomography
PICO	Population, Intervention, Comparison, Outcome
PPV	Positive Predictive Value
PRISMA	Preferred Reporting Items for Systematic Reviews and Meta-Analyses
RCT	Randomized Controlled Trial
RoB	Risk of Bias
RRR	Resistive Reserve Ratio
RV	Right Ventricle/Right Ventricular
SBI	Silent Brain Infarction
SPECT	Single Photon Emission Computed Tomography
SR/MA	Systematic Review/Meta-Analysis
SSFP	Steady-State Free Precession
STEMI	ST-Elevation Myocardial Infarction
SYNTAX	Synergy Between Percutaneous Coronary Intervention with Taxus and Cardiac Surgery (score)
T1	T1 relaxation time mapping (MRI sequence)
T2	T2 relaxation time mapping (MRI sequence)
T2-STIR	T2-weighted Short Tau Inversion Recovery
TRT	Thermodilution-derived Temperature Recovery Time
WHCA	Whole-Heart Coronary Angiography (MR-based)
